# Evaluation of Chest and Abdominal Injuries in Trauma Patients Hospitalized in the Surgery Ward of Poursina Teaching Hospital, Guilan, Iran

**DOI:** 10.5812/atr.7672

**Published:** 2013-02-01

**Authors:** Hossein Hemmati, Ehsan Kazemnezhad-Leili, Zahra Mohtasham-Amiri, Ali Asghar Darzi, Ali Davoudi-Kiakalayeh, Anoush Dehnadi-Moghaddam, Leila Kouchakinejad-Eramsadati

**Affiliations:** 1 Department of Surgery, Guilan Road Trauma Research Center, Guilan University of Medical Sciences, Rasht, IR Iran; 2 Department of Biostatistics, Guilan Road Trauma Research Center, Guilan University of Medical Sciences, Rasht, IR Iran; 3 Department of Preventive and Social Medicine, Guilan Road Trauma Research Center, Guilan University of Medical Sciences, Rasht, IR Iran; 4 Department of Surgery, Babol University of Medical Sciences, Babol, IR Iran; 5 Guilan Road Trauma Research Center, Guilan University of Medical Sciences, Rasht, IR Iran; 6 Department of Anesthesiology, Guilan Road Trauma Research Center, Guilan University of Medical Sciences, Rasht, IR Iran

**Keywords:** Abdomen, Chest, Traffic Accident, Wounds and Injuries

## Abstract

**Background:**

Trauma, especially chest and abdominal trauma are increasing due to the growing number of vehicles on the roads, which leads to an increased incidence of road accidents. Urbanization, industrialization and additional problems are the other associated factors which accelerate this phenomenon. A better understanding of the etiology and pattern of such injuries can help to improve the management and ultimate the outcomes of these patients.

**Objectives:**

This study aimed to evaluate the patients with chest and abdominal trauma hospitalized in the surgery ward of Poursina teaching hospital, Guilan, Iran.

**Patients and Methods:**

In this cross-sectional study, the data of all chest and abdominal trauma patients hospitalized in the surgery ward of Poursina teaching hospital were collected from March 2011 to March 2012. Information about age, gender, injured areas, type of injury (penetrating or blunt), etiology of the injury, accident location (urban or rural) and patients' discharge outcomes were collected by a questionnaire.

**Results:**

In total, 211 patients with a mean age of 34.1 ± 1.68 years was entered into the study. The most common cause of trauma was traffic accidents (51.7%). Among patients with chest trauma, 45 cases (35.4%) had penetrating injuries and 82 cases (64.6%) blunt lesions. The prevalence of chest injuries was 35.5% and rib fractures 26.5%. In chest injuries, the prevalence of hemothorax was 65.3%, pneumothorax 2.7%, lung contusion 4% and emphysema 1.3%, respectively. There were 24 cases (27.9%) with abdominal trauma which had penetrating lesions and 62 cases (72.1%) with blunt lesions. The most common lesions in patients with penetrating abdominal injuries were spleen (24.2%) and liver (12.1%) lesions. The outcomes of the patients were as follow: 95.7% recovery and 4.3% death. The majority of deaths were observed among road traffic victims (77.7%).

**Conclusions:**

Considering the fact that road-related accidents are quite predictable and controllable; therefore, the quality promotion of traumatic patients' care, and the road safety should be noted as problems associated with public health.

## 1. Background

Along with industrialization, scientific and technological development in the recent century, trauma and its complications has become an important issue, as it is one of the most prevalent causes of fatalities and morbidity worldwide, particularly in developing and developed countries (1). In Iran, trauma is the fourth cause of mortality in individuals under 45 years of age, the third main cause of death in all age groups, and it is the second cause of death among young people (2). Despite remarkable developments in trauma management, it is still a significant cause of mortality. Therefore, trauma is a major hazard in every society, which affects health, economic and social indicators (3). Trauma, abdominal and chest trauma in particular, have been increasing steadily following a surge in vehicle numbers that have led to an increase in road accidents, along with growing urbanization and industrialization related problems (4). Based on WHO (World Health Organization) predictions, road traffic accidents will become the second cause of premature death in the world by 2020 (5). Chest trauma is an important cause of mortality and morbidity, and it stands in the third global place after cancer and cardiovascular diseases (4, 6). It has been declared that 20 - 25% of fatalities in trauma patients are related to chest injuries (7). Furthermore, abdominal injury is very common in trauma victims (8). It has been determined that 20% of traumas due to road accidents occur in the abdomen (2).

## 2. Objectives

The present research aimed to study chest and abdominal trauma patients who attended Poursina teaching hospital, Guilan, Iran. Poursina teaching hospital is the main trauma center located in the north of Iran and it is responsible for the treatment of trauma patients every day, thus a better understanding of the etiology and pattern of these types of injuries could improve the management and final outcomes of these patients.

## 3. Patients and Methods

In a cross-sectional study, the information of 211 (88.2% male and 11.2% female) chest and abdominal trauma patients in the general surgery ward of the Poursina teaching hospital, were surveyed from March 2011 to March 2012. All trauma patients hospitalized with penetrating or blunt chest and abdominal trauma, with or without other organ damage, were entered into the study. Those without the required information in their hospital records were excluded. Information about a patient’s age, gender, injured areas, type of injury (blunt or penetrating), etiology of injury, location of accident (urban and rural), and discharge outcomes were collected by a questionnaire. Data were analyzed with descriptive statistics using SPSS (v.16, SPSS Inc., Chicago, Illinois). Means and percentages were used to describe the data. A χ2 test was used to compare the frequency distribution of chest and abdomen trauma based on qualitative variables, and an independent student's t-test was used to compare the quantitative variables such as length of stay in hospital (LOS) in terms of blunt and penetrating trauma. The significance level in this study was considered as P < 0.05 and the tests were two-tailed.

## 4. Results

Out of the 692 hospitalized trauma patients, 211 had the inclusion criteria and were entered into the study. Mean age of the patients was 34.1 ± 1.68 years (4 - 75 y). Young adults (16 - 59 y) had the highest rate (83.9%). A total of 211 patients were male (88.2%) and 25 female (11.8%). According to the causes of the trauma, it was shown that road traffic accidents (n = 109, 51.7%) and assaults (n = 68, 32.2%), were the most prevalent causes of trauma (Table 1). In chest and abdominal trauma due to road accidents; motor cyclists (n = 25, 22.9%), pedestrians (n = 18, 16.5%), followed by vehicle drivers (n = 15, 13.8%), were the most frequently injured groups.

**Table 1:** Demographic Characteristics and Types of Injury Among Trauma Patients

Data Category	No. (%)
**Gender**	
Male	186 (88.2)
Female	25 (11.8)
**Age, y**	
1 - 15	11 (5.2)
16 - 59	177 (83.9)
≥ 60	23 (10.9)
**Injury Mechanism**	
Motor vehicle accidents	109 (51.7)
Falling	18 (8.5)
Assault	68 (32.2)
Suicide	6 (2.8)
Other unintentional	10 (4.7)
**Injury Type**	
Abdominal injury	
**Blunt**	62 (72.1)
**Penetrating**	24 (27.9)
Thoracic injury	
**Blunt**	82 (64.6)
**Penetrating**	45 (35.4)
**Outcome**	
Death	9 (4.3)
**Survival**	202 (95.7)

The incidence of trauma in urban and rural areas was 47.4% and 52.6%, respectively. In the latter, the prevalence of blunt injuries was greater, while in the former, penetrating abdominal and chest injuries were the most frequent type of injury. The frequency distributions of abdominal injuries based on the accident location were statistically significant (P < 0.035). Blunt chest trauma constituted 47.8% and 83.3% of all injuries in urban and rural areas, respectively, and penetrating chest injuries were 52.2% and 16.7%, respectively. There was a significant difference in the frequency distribution of the chest injuries in terms of accident location (P < 0.001). In total, 127 patients (60.2%) were observed with chest trauma and 86 (40.8%) with abdominal trauma, and only two patients had both abdominal and chest traumas. Among the patients with chest trauma, 45 (35.4%) had penetrating and 82 (64.6%) blunt lesions. In patients with chest trauma, the prevalence of chest damage was 35.5%, and rib fractures 26.5%. In chest trauma, hemothorax had a 65.3% prevalence, pneumothorax 2.7%, lung contusion 4%, and emphysema 1.3% (Table 2). There were 24 (27.9%) abdominal trauma patients who had a penetrating trauma and 62 (72.1%) with blunt lesions. The most common injuries were spleen (24.2%), and liver (12.1%) injuries (Table 3). The average length of hospitalization was 10.78± 12.53 days in patients with blunt chest trauma, and 5.74 ± 3.73 days in patients with a penetrating chest injury (P < 0.002). The average figures were 9.5 ± 9.79 and 4.6 ± 2.36 days, in patients with blunt and penetrating abdominal trauma, respectively, which highlights the length of hospitalization time required as being twice the length in patients with blunt trauma (P < 0.001). The outcome of patients at discharge was 95.7% recovery and 4.3% mortality. Overall, 4.9% of patients with blunt and 2.2% with penetrating chest traumas died. Moreover, all of the abdominal trauma patients who died had blunt lesions (8.1%). The greatest number of mortalities was seen in patients with chest and abdominal traumas derived from road traffic injuries (77.7%).

**Table 2:** Pattern of Chest Injuries

Pattern	No. (%)	Blunt	Penetrating	*P* value
**Hemothorax**	49 (65.3)	32	17	0.44
**Pneumothorax**	2 (2.7)	1	1	0.999
**Contusion**	3 (4)	3	0	0.250
**Emphysema**	1 (1.3)	1	0	-a
**Hemothorax and Pneumothorax**	8 (10.7)	5	3	0.727
**Hemothorax and Emphysema**	7 (9.3)	7	0	0.16
**Pneumothorax and Emphysema**	3 (4)	1	2	0.999
**Hemothorax, Pneumothorax and Emphysema**	2 (2.7)	2	0	0.500

^a^Inadequate sample

**Table 3:** Pattern of Abdominal Injuries

Pattern	No. (%)	Blunt	Penetrating	*P* value
**Spleen **	16 (24.2)	15	1	- a
**Liver**	8 (12.1)	7	1	0.70
**Kidney**	2 (3)	2	0	0.500
**Mesentery**	4 (6.1)	3	1	0.625
**Rectum**	11 (16.6)	6	5	0.999
**Jejunum-ileum**	7 (10.6)	5	2	0.453
**Peritoneum**	3 (4.5)	3	0	0.250
**Colon**	3 (4.5)	1	2	0.999
**Diaphragm**	1 (1.5)	1	0	- a
**Vagina**	2 (3)	2	0	0.500
**Jejunum-ileum and Spleen**	1 (1.5)	1	0	- a
**Liver and Ileum**	1 (1.5)	1	0	- a
**Liver and Spleen**	2 (3)	2	0	0.500
**Liver and Kidney**	1 (1.5)	1	0	- a
**Spleen and Pancreas**	1 (1.5)	1	0	- a
**Liver, Diaphragm and Retroperitoneum**	1 (1.5)	1	0	- a
**Spleen and Pancreas and Liver**	1 (1.5)	1	0	- a

^a^Inadequate sample

## 5. Discussion

Trauma is usually considered as the main cause of mortality and morbidity in individuals between 1 - 44 years (9). In an ongoing study, the mean age of patients was 34.1 years and the highest frequency was seen between 16 - 59 years-of-age, which shows that chest and abdominal traumas are more common in young people. In other words, trauma affects the quality of life and life itself, in a group that consists of the most efficient in society, and this brings about huge economic losses. The most at-risk chest and abdominal trauma group was men, giving rise to a ratio of women to men of 1/7.44, this is expected to occur as a result of the current social and economic state of Iran and the fact that most men work away from their home. Similar results have also been reported in previous studies. In a study on hospitalized trauma patients at the Poursina teaching hospital, most of the patients were in the age range 20 - 44 years and the ratio of trauma affected men was 3.6 times more than women (10). In a study by Ahmadi et al., most of the chest traumatic patients were adult men with a mean age of 35 years old and mode of 30, and road accidents were the most prevalent cause of trauma (1). Gad et al. reported that most of the abdominal trauma patients were men and motor vehicle accidents were the main cause of abdominal trauma followed by falling or assault in second place (3). In a study by Baradaran et al. it was revealed that most patients with penetrating abdominal trauma were men (89.9%) and the main cause of the penetrating trauma was a knife wound. Based on their study, men had this type of trauma 22 times more often than women, most of whom were in age range of 15 - 44 years old. Moreover, the colon and liver were the organs that sustained the most damage following a trauma (9). In the present study, as in previous studies on road accidents, assaults and falling were the main causes of chest and abdominal trauma. Among road accidents leading to trauma, motorists, followed by pedestrians and passengers were the most at-risk groups. In the majority of previous studies, road accidents were mentioned as the main cause of trauma (3, 8, 11). Comparing the incidence of chest and abdominal trauma in rural and urban areas revealed that the incidence of this type of trauma was higher in rural areas compared with urban ones. Furthermore, the prevalence of blunt compared with penetrating trauma was greater in rural regions. Jansen et al. have gained results similar to ours and accordingly blunt abdominal trauma is more prevalent in rural areas (12). Similar studies have been reported in a study by Hemmila et al. while this type of population distribution was not observed in a study by Gad et al. (4, 13). The highest number of trauma patients in the present research had chest trauma. Most of the damage resulted from blunt trauma. The greater prevalence of blunt trauma damage compared with penetrating injuries was also reported in patients with chest and abdominal trauma in previous studies (1, 3, 8, 11, 14). In patients with penetrating abdominal lesions, the most common were liver and spleen damage. Similar results were described by Smith et al. and Isenhour et al. (11, 15). According to a study by Godbole and Stringer (16), most internal damage of the abdomen occurred in the spleen. In patients with chest trauma, lung tissue damage and then rib fractures were the most common injuries. Among injuries to the lung, hemothorax was the condition most often manifested. Ali Khan et al. in their studies on 114 patients with chest trauma declared that hemothorax and pneumothorax were the most prevalent lesions in these patients (4). Similar results were witnessed in the Yalçinkaya et al. study (17). The length of hospitalization in patients with blunt chest and abdominal trauma was almost twice that seen in penetrating trauma victims. Moti et al. examining the length of hospitalization in patients with blunt abdominal trauma, evaluated the minimum and maximum time of hospitalization as being between 1 to 44 days with an average of 8.79 days ± SD = 8.99 (2). In a study conducted in the Al-Zahra hospital, Isfahan, Iran, the average length of hospitalization in cases with penetrating trauma was 6.9 ± 3.2 and in those with blunt trauma was 7.37 ± 7.1 days (18). Rate of mortality in this series of patients was 4.3%. Most of the deaths were seen in patients with chest and abdominal trauma resulting from road traffic accidents which seems to be due to the fact that the Poursina hospital is a special center with the most referrals from victims of road traffic accidents in the northern region of Iran. In some previous studies, knife wounds (9) and in others, motor car accidents (10, 14), were mentioned as being the main cause of death. The majority of patients died due to blunt abdominal and chest injuries. Similar results have been reported in a study by Haratian et al. (14). The results showed that men were more often exposed to chest and abdominal trauma than women. In addition, the prevalence of blunt injuries was greater in the patients under study. In urban and rural areas, chest and abdominal blunt damage were more frequent than penetrating injuries. In addition to promoting quality of care in trauma patients, given that a significant proportion of abdominal and chest trauma and deaths were due to injuries caused by road accidents, and the fact that events of this type can be predicted and controlled, road safety issues should be noted as a matter related to public health and considered as such. Thus, local and national information is considered to be an important resource for road safety planning and management decisions.
